# Effects of urbanization on host-pathogen interactions, using *Yersinia* in house sparrows as a model

**DOI:** 10.1371/journal.pone.0189509

**Published:** 2017-12-27

**Authors:** Lieze Oscar Rouffaer, Diederik Strubbe, Aimeric Teyssier, Noraine Salleh Hudin, Anne-Marie Van den Abeele, Ivo Cox, Roel Haesendonck, Michel Delmée, Freddy Haesebrouck, Frank Pasmans, Luc Lens, An Martel

**Affiliations:** 1 Department of Pathology, Bacteriology and Avian Diseases, Faculty of Veterinary Medicine, Ghent University, Merelbeke, Belgium; 2 Department of Biology (Terrestrial Ecology Unit), Faculty of Sciences, Ghent University, Ghent, Belgium; 3 Department of Biology, Faculty of Science, Antwerp University, Antwerp, Belgium; 4 Department of Biological Sciences, Faculty of Science & Mathematics, Universiti Pendidikan Sultan Idris, Tanjong Malim, Perak, Malaysia; 5 Microbiology Laboratory, AZ Sint Lucas Ghent, Ghent, Belgium; 6 Institute of Experimental and Clinical Research, Université Catholique de Louvain, Brussels, Belgium; Institut Pasteur, FRANCE

## Abstract

Urbanization strongly affects biodiversity, altering natural communities and often leading to a reduced species richness. Yet, despite its increasingly recognized importance, how urbanization impacts on the health of individual animals, wildlife populations and on disease ecology remains poorly understood. To test whether, and how, urbanization-driven ecosystem alterations influence pathogen dynamics and avian health, we use house sparrows (*Passer domesticus*) and *Yersinia* spp. (pathogenic for passerines) as a case study. Sparrows are granivorous urban exploiters, whose western European populations have declined over the past decades, especially in highly urbanized areas. We sampled 329 house sparrows originating from 36 populations along an urbanization gradient across Flanders (Belgium), and used isolation combined with ‘matrix-assisted laser desorption ionization- time of flight mass spectrometry’ (MALDI-TOF MS) and PCR methods for detecting the presence of different *Yersinia* species. *Yersinia* spp. were recovered from 57.43% of the sampled house sparrows, of which 4.06%, 53.30% and 69.54% were identified as *Y*. *pseudotuberculosis*, *Y*. *enterocolitica* and other *Yersinia* species, respectively. Presence of *Yersinia* was related to the degree of urbanization, average daily temperatures and the community of granivorous birds present at sparrow capture locations. Body condition of suburban house sparrows was found to be higher compared to urban and rural house sparrows, but no relationships between sparrows’ body condition and presence of *Yersinia* spp. were found. We conclude that two determinants of pathogen infection dynamics, body condition and pathogen occurrence, vary along an urbanization gradient, potentially mediating the impact of urbanization on avian health.

## Introduction

With growing human populations, cities are expanding rapidly and urbanization represents one of the most intense anthropogenic modifications of natural systems, strongly affecting species, communities and ecosystems [1,2]. The direction and strength of responses of bird species to urbanization is function of their life-history strategies [3]. This has led to the ‘biotic homogenization’ of urban bird communities [4], i.e. whereby the latter become gradually dominated by a limited number of ‘urban exploiter’ species, such as house sparrows *(Passer domesticus)* [5]. Studies focussing on how avian communities respond to urbanization find that bird species richness [4,6,7] and population densities [6] are often highest at intermediate levels of urbanization. However, although several authors have addressed the effects of urbanization on avian stress levels and body condition (e.g. [8–11]), how individuals of urban exploiters successfully cope with urban environments, remains poorly understood.

How urbanization affects disease ecology, wildlife-pathogen interactions and animal health remains particularly underexplored, despite its potential effect on ecological and evolutionary mechanisms driving population dynamics [12–16]. In addition, wildlife is increasingly being recognized as an important vector, or potentially even reservoir, for various human diseases [17], such as yersiniosis, the third most commonly reported bacterial zoonotic disease in Europe in 2013 [18]. In humans, yersiniosis is most frequently caused by *Yersinia enterocolitica* biotype (BT) 1B and 2–5 and to a lesser extent by *Y*. *pseudotuberculosis* [18,19]. In passerines, the facultative pathogen *Y*. *pseudotuberculosis* is the most probable etiologic agent of yersiniosis, which typically has an acute enteric disease progression [20–22], but has on several occasions been isolated from apparently healthy birds [23,24]. Although it is possible that these birds were in the incubation phase of the disease, it has been speculated that wild-ranging birds maintain the bacteria at low level, developing acute disease when subjected to stressful conditions [24]. Yet, the potential existence of subclinical effects on avian health and body condition remains a gap in our knowledge.

So far only few studies have focused on the combination of differential pathogen exposure along urbanization gradients and the effects on the body condition of their avian hosts (e.g. [15,25,26]). With respect to *Yersinia*, their psychrotolerant nature [27] potentially renders these bacteria susceptible to microclimate differences (e.g. heat island effect) between urbanized and rural areas [28]. In addition, the distinct metabolic flexibility of various *Yersinia* species [29] may affect environmental survival and persistence, enhancing the survival of the less pathogenic environmental strains with higher metabolic capacity compared to the more pathogenic strains which are metabolically more constrained and are more dependent on the presence of suitable hosts. Depending on the pathogen-suitability of the hosts, higher host diversity or density may both reduce or amplify the bacterium-load in the environment [12], and hence, the faeco-oral transmission of pathogenic *Yersinia* species. Not only can *Yersinia* affect birds’ health, but vice versa, avian health, related to stress and estimated by body condition [30], could affect the excretion of pathogens in the environment [31,32].

In order to gain more insights into urban wildlife-disease ecology, we assessed the prevalence of an important zoonotic and avian pathogen (i.e. *Yersinia* spp.) in house sparrows along an urbanization gradient. House sparrows constitute an adequate study species as they inhabit rural, suburban and urban areas, they are considered to be very sedentary, and they have experienced severe population declines over the last decades, especially in urban centres [33–36]. We evaluated how urbanization and the local community of granivorous birds impact on house sparrows’ body condition and on the presence of *Yersinia* spp. in their faeces, in combination with the two-way host-pathogen interaction, taking into account temperature and time of sparrow capture during sampling.

## Material and methods

### House sparrow sampling and environmental data

**Fig 1:**
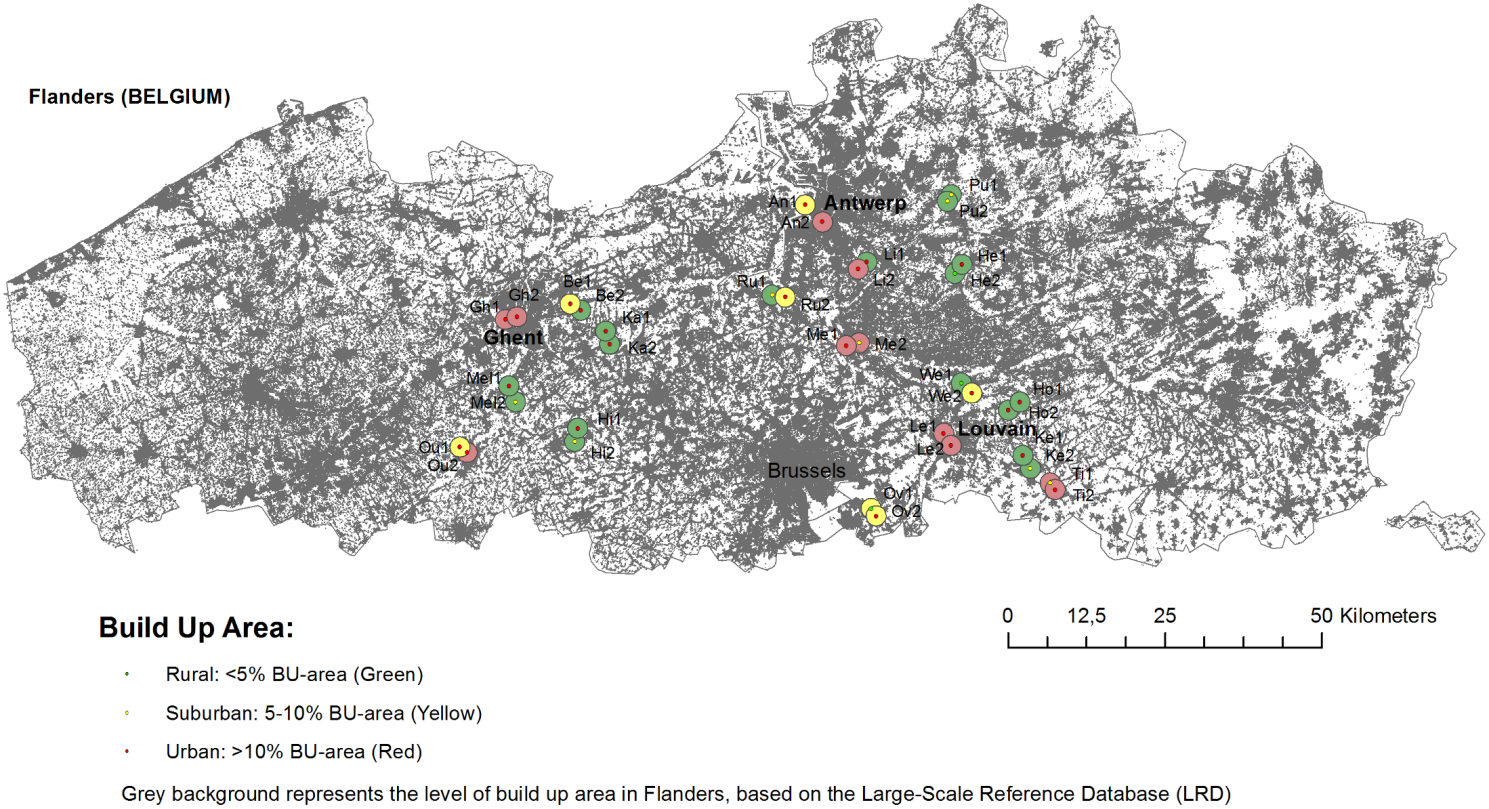
House sparrow populations clustered around the cities of Ghent, Antwerp and Leuven.

Since disease outbreaks most often occur during winter [21,23,37], faecal samples from 329 house sparrows were collected during two consecutive sampling periods, i.e. 3 October till 20 December 2013 (‘autumn’) and 10 January until 28 March 2014 (‘winter-early spring’), respectively. Sampled house sparrows originated from 36 populations located in 11 ‘urban’, 7 ‘suburban’ and 18 ‘rural’ regions (details on urbanization levels are given in the supporting information: S1 Table) clustered pairwise around the Flemish cities of Ghent, Antwerp and Leuven (Fig 1), every population being sampled at least once per sampling period.

The sampling protocol is as described in [38]. Upon capture, each individual was ringed, sexed, weighed (±0.01g: digital balance) and their tarsus length was measured (±0.01mm: digital calliper). To quantify sparrows’ body condition, we applied the scaled-mass index (SMI), which adjusts the mass of all individuals to that which they would have obtained if they all had the same body size, using the equation of the linear regression of ln-body mass on ln-tarsus length estimated by type-II (standardized major axis; SMA) regression [30]. Two outliers were present in the data (i.e. |standardized residuals| > 3), these two observations were not considered for deriving the SMI relationship. The regression slope was 1.50 and average tarsus length was 18.8 mm. We thus calculated the SMI as body mass x (18.8/tarsus length)^1.50 [30]. House sparrows are considered species of Least Concern on the ‘IUCN Red List of Threatened Species’ [39] and all people involved in the sampling were holders of a scientific ringing certificate issued annually by the Agency for Nature and Forest. The sparrows were captured on private land for which oral permission was granted by the respective land owners. All trapping and sampling protocols were approved by the Ethical Committee VIB Ghent site (EC2013-027).

As environmental predictors, we considered the degree of urbanization, the average air temperatures at the day of sampling and the presence of other granivorous birds. In order to quantify the degree of urbanization at sampling sites, the level of built-up area (BU) was calculated in circular plots around each trapping site based on the very high resolution (i.e. 0.15m pixels) ‘Large-scale Reference Database’ (LRD) GIS layers [40,41], both at a local ‘home-range’ scale (using a 100 m radius around the capture site) and at a ‘landscape’ scale (using a 1600 m radius around capture site, thereby excluding the 100 m radius of the home range scale) [35,42]. The extent of the home-range scale was based on radio-telemetric observations of habitat use by Flemish house sparrows [8] and represents the extent of daily foraging movements. The landscape scale was based on population genetic estimates [35] and reflects the average distance at which sparrow populations can genetically be considered independent from each other. To ensure a more natural environment for the lowest urbanization class, we only selected plots comprising >20% of ecologically valuable areas, as described by the Flemish Governments’ Biological Valuation Map [43]. Urbanization at the home-range scale was modelled as a continuous variable (range 1.72–55.04% BU area), while at the landscape scale, it was modeled as class variable, i.e. ‘rural’ (<5% built-up area), ‘suburban’ (5–10%) or ‘urban’ (>10%) [44]. Average daily temperatures were derived from the nearest located weather station and were provided by the Belgian Royal Meteorological Institute (RMI). For every house sparrow population under study, a granivore-index was calculated, i.e. indicating the degree to which a local bird assemblage is dominated by granivorous species which could, through similar foraging strategies, have a higher potential of exchanging enteropathogenic bacteria through the faeco-oral transmission route [45–48]. Since conducting bird surveys during sampling was not feasible because of logistic reasons, we relied on data collected during the most recent Flemish breeding bird atlas [49] whereby the Flemish region was divided in a grid of 5km x 5km. Within each of these squares, bird surveyors were instructed to carry out two one-hour long visits to sets of eight fixed 1km x 1km squares in order to arrive at a list of breeding bird species (see [50] for details). For each sparrow sampling site, we determined the closest (5x5 km) grid cell sampled by the breeding bird atlas (using Euclidean distance) and extracted the species list for that grid cell. Each bird species present was assigned a ‘granivore score’, varying from 0 to 1, based on bird diets as mentioned in [51]. Following [3], scoring was as follows: 0 = no grains, 0.1 = occasionally grains, 0.5 = frequently grains, 1 = almost exclusively grains. In order to obtain an overall ‘granivore-index’ for each sparrow sampling site, we summed the granivore-scores of all birds present in a grid cell and divided this sum by the total number of bird species present.

### *Yersinia* isolation and identification

Faecal samples were subjected to a cold enrichment procedure in combination with an alkali (KOH) treatment as described in [52]. This isolation method has previously been demonstrated to be the most successful method for the isolation of *Y*. *pseudotuberculosis* and *Y*. *enterocolitica*, even when only small numbers of bacteria are present in a sample [24,53]. All the colonies suspicious for *Yersinia* were biochemically tested at 30°C using Kligler (Oxoid, Ltd), Aesculine (Oxoid, Ltd.) and Urea (Oxoid, Ltd), before performing MALDI-TOF MS (Matrix-Assisted Laser Desorption Ionization-Time of Flight Mass Spectrometry) at the Department of Clinical Microbiology, Laboratory Medicine, AZ Sint-Lucas in Ghent. Every MALDI-TOF assigned-*Y*. *enterocolitica* and *Y*. *pseudotuberculosis* was subjected to virulence PCR on chromosomal- (*ail*, *ystA*, *ystB*, *inv*) and plasmid-borne- (*virF*) virulence genes, according to the PCR-protocol and primers used by [19]. *Yersinia pseudotuberculosis* (22.36a), human pathogenic *Y*. *enterocolitica* 4/O:3 (75.55b) and *Y*. *enterocolitica* BT1A (FAVV208) were used as positive controls. If virulence genes were detected, *Y*. *pseudotuberculosis* isolates were serotyped at the National Reference Center *Yersinia* (IREC).

Although PCR on the combination of chromosomal- and plasmid-borne virulence genes and MALDI-TOF MS has previously been used for the identification of (enteropathogenic) *Y*. *enterocolitica* and *Y*. *pseudotuberculosis* [19,52,54–57], the accurate species identification of the latter technique is highly dependent on the validation of the reference library used to identify the bacterial isolates, resulting in high sensitivity and specificity for the validated species [55,56,58]. This validation was performed for *Y*. *pseudotuberculosis* and *Y*. *enterocolitica* on the Bruker Daltonik MALDI Biotyper at the Department of Clinical Microbiology [59], but not for other *Yersinia* species. As such, the *Yersinia* species other than *Y*. *enterocolitica* and *Y*. *pseudotuberculosis* were not identified up to species level and are included in the statistics as “*Yersinia* species”.

### Statistical analyses

First, in order to test whether *Yersinia* spp. prevalence was related to the degree of urbanization and presence of possible host species (expressed by the granivore-index), we applied Generalized Linear Mixed Models (GLMM) [60,61] with a binomial error distribution, using the R ‘lme4’, ‘lmerTest’, ‘Hmisc’, ‘plyr’ and ‘effects’ packages [62–66]. Degree of urbanization at home-range and landscape scales (and the two-factor interaction), granivore-index, daily average temperature, sex and host SMI were modelled as fixed effects, while sampling period was modeled as a random effect using the glmer command (S1 Protocol; S1 and S2 Datasets). To account for possible spatial autocorrelation in *Yersinia* prevalence, latitude and longitude of sampling locations were included as fixed effects [67]. Separate models were run to identify factors influencing the distribution of “*Y*. *enterocolitica”*, *“Y*. *pseudotuberculosis”*, “*Yersinia* spp. other than *Y*. *enterocolitica* and *Y*. *pseudotuberculosis”*. We applied a model selection procedure based on Akaike’s Information Criterium AIC [68] and calculated AICc values for all possible models, using the R MuMIn package [69]. Models were ranked based on their AICc values, and the relative importance of variables was assessed by summing the AICc weights of all models in which the variable under consideration was included. Important variables are characterized by a high AICc weight (i.e. >0.5) and model-averaged estimates that are higher than their standard errors [70].

Second, to test whether host SMI was impacted by *Yersinia* spp. along the urbanization level, we applied a linear mixed model (LMM) using a Gaussian error distribution, including presence/absence of *Y*. *enterocolitica*, *Y*. *pseudotuberculosis* or other *Yersinia* spp., degree of urbanization at home-range and landscape scales (and two-factor interaction), sex, granivore-index, daily average temperature and time (hour) of capture as fixed effects, and sampling period as random effect, using the same packages as for the GLMM, and the lmer-function (S1 Protocol; S1 and S2 Datasets). Model residuals were normally distributed (Shapiro-Wilk W > 0.95). Since the AIC-weight of the two-way interaction (see higher) was low (<0.5) for all the GLMM and LMM analyses, models were rerun without interaction to obtain final AIC-weights. All analyses were conducted in R [71].

## Results

In total, 329 house sparrows (143 females, 186 males) were captured from rural (51%), suburban (14%) and urban habitats (35%) (S1 Table). All individuals, with the exception of one bird which was diagnosed with pox-virus [38], were apparently healthy. *Yersinia* species were isolated from 59% (193/329) of the examined hosts with *Y*. *enterocolitica* being the most commonly isolated *Yersinia* species, isolated from 31% (103/329) of the individuals (S1 Table). Except for the *ystB*-gene, identified in 92 (89%) of the *Y*. *enterocolitica* isolates, none of the isolates harbored the examined virulence genes. *Y*. *pseudotuberculosis* was recovered from 2% (8/329) of the hosts (S1 Table). With four isolates, serotype I was the most encountered serotype. Two isolates were identified as serotype II and two as serotype III and V respectively. All the isolates, apart from both serotype II isolates, originated from different house sparrow populations. Except for serotype III and V, which did not possess the *virF* plasmid-borne virulence gene, both the *inv-* and *virF*-gene were detected in the different serotypes. *Yersinia* species, other than *Y*. *enterocolitica* and *Y*. *pseudotuberculosis* were isolated from 41% (134/329) of the house sparrows. In total 51 house sparrows harbored multiple *Yersinia* species in their faeces.

When testing for drivers of different *Yersinia* spp. presence in house sparrow’ faeces, AIC-based model averaging appointed different variables as important explanatory variables, depending on the *Yersinia* species tested (Table 1). Presence of *Y*. *pseudotuberculosis* was best explained by the granivore-index, for which a positive relationship was observed (AIC-weight: 0.90, estimate ± standard error: 1.18±0.59; Tables 2 and 3). In addition, landscape-level urbanization influences *Y*. *pseudotuberculosis* distribution: compared to rural habitats, this species tends to be most prevalent in suburban habitats, and to a lesser extent in urban habitats (AIC-weight: 0.61, estimate: 2.83±1.35 and 1.95±1.08 resp.; Tables 2 and 3). No strong evidence for an effect of host SMI, sex, daily average temperature and home-range level factor on presence of *Y*. *pseudotuberculosis* was evident (AIC-weights <0.5; Table 2). Variables best explaining the presence of *Y*. *enterocolitica* were, in order of importance, daily average temperature, the granivore-index, the percentage of built-up area at the home-range scale and, to a lesser extent, at the landscape scale. *Yersinia enterocolitica* was negatively correlated to daily average temperatures (AIC-weight: 1.00, estimate: -0.68±0.17), to the granivore-index (AIC-weight: 0.92, estimate: -0.39±0.15) and to the percentage of built-up area at home-range level (AIC-weight: 0.75, estimate: -0.32±0.16) (Tables 2 and 3). At the landscape level, the prevalence of *Y*. *enterocolitica* tends to be lower in suburban house sparrows, compared to the urban and (to a lesser degree) to the rural birds (AIC-weight: 0.59, estimate: 0.96±0.48 and 0.79±0.49 resp.; Table 3). Nor the SMI, nor the sex influenced *Y*. *enterocolitica* prevalence (AIC-weight: <0.5; Table 2). Presence of other *Yersinia* species was best explained by the average daily temperature (AIC-weight: 0.92, estimate: -0.31±0.12), to which it was negatively related, and by the home-range level (AIC-weight: 0.67, estimate: -0.21±0.12), as *Yersinia* species tended to be less prevalent in more urbanized core habitats (Tables 2 and 3). After accounting for the effect of time of capture (AIC weight: 0.76, 0.06±0.0.3), we found that sparrow body condition (i.e. SMI) was correlated to landscape-level urbanization (AIC weight: 0.64) (Tables 1–3). The SMI was generally higher for suburban house sparrows compared to either urban (estimate: -0.43±0.18) or rural house sparrows (estimate: -0.27±0.17) (Table 3). Specifically, suburban sparrows were on average 3% heavier than urban birds and 2% than rural sparrows. Presence of *Y*. *enterocolitica*, *Y*. *pseudotuberculosis* or other *Yersinia* species, average daily temperatures, sex, granivore-index or home-range level urbanization did not affect hosts SMI (all variable AIC-weights <0.5; Table 2).

**Table 1:** Best models using AIC-based model selection for *Y*. *pseudotuberculosis*, *Y*. *enterocolitica*, other *Yersinia* species and Scaled Mass Index as respective response variables.

Response variable: explanatory variables	Log(L)	AIC	ΔAIC	weight
***Y*. *pseudotuberculosis***: Granivore-index, Urbanization (landscape level)	-32.21	78.76	0.00	0.64
***Y*. *enterocolitica***: Average temperature, Granivore-index, Urbanization (home range level), Urbanization (landscape level)	-185.47	389.50	0.00	0.42
**Other *Yersinia* species**: Average temperature, Urbanization (home range level)	-218.75	449.75	0.00	0.60
**SMI**: Time of capture, Urbanization (landscape level)	-465.15	946.74	0.00	0.55

**Table 2:** Variable importance after model-averaging in order to explain the presence of *Y*. *pseudotuberculosis*, *Y*. *enterocolitica* and other *Yersinia* species and the SMI of the host.

	*Y*. *pseudotuberculosis*	*Y*. *enterocolitica*	Other *Yersinia* species	SMI
Granivore-index	**0.90**	**0.92**	0.32	0.49
Urbanization (landscape level)	**0.61**	**0.59**	0.16	**0.64**
Urbanization (home range level)	0.38	**0.75**	**0.67**	0.48
Average temperature	0.39	**1.00**	**0.92**	0.27
Scaled Mass Index	0.26	0.30	0.35	NA
Sex	0.44	0.26	0.44	0.38
Time of Capture	NA	NA	NA	**0.76**
*Y*. *pseudotuberculosis*	NA	NA	NA	0.26
*Y*. *enterocolitica*	NA	NA	NA	0.39
Other *Yersinia* species	NA	NA	NA	0.38

NA (not applicable)

**Table 3:** Parameter estimates and standard deviation for response variables: *Y*. *pseudotuberculosis*, *Y*. *enterocolitica*, other *Yersinia* species and SMI (shown in Table 1).

Parameters for *Y*. *pseudotuberculosis*	Estimate ± SE
Granivore-index	1.18±0.59
Urbanization landscape (Suburban)^a^	2.83±1.35
Urbanization landscape (Urban)^a^	1.95±1.08
**Parameters for *Y*. *enterocolitica***	
Average temperature	-0.68±0.17
Granivore-index	-0.39±0.15
Urbanization home range	-0.32±0.16
Urbanization landscape (Urban)^b^	0.96±0.48
Urbanization landscape (Rural)^b^	0.79±0.49
**Parameters for other *Yersinia* species**	
Average temperature	-0.31±0.12
Urbanization home range	-0.21±0.12
**Parameters for SMI**	
Time of capture	0.06±0.0.3
Urbanization landscape (Urban)^b^	-0.43±0.18
Urbanization landscape (Rural)^b^	-0.27±0.17

^a^ Urbanization within 1600m radius is compared to the Rural habitat

^b^ Urbanization within 1600m radius is compared to the Suburban habitat

## Discussion

A high prevalence of *Yersinia* was demonstrated in the faeces of the examined house sparrows, of which most isolates belonged to *Y*. *enterocolitica* and only a small percentage to *Y*. *pseudotuberculosis*. These results are in agreement with previous reports using cold enrichment methods [24,31,37]. Apart from the *ystB*-gene, which was demonstrated in most of the *Y*. *enterocolitica* isolates and is associated with biotype 1A [19,54], no human-pathogenic *Y*. *enterocolitica* biotype was recovered from our house sparrows. In humans, controversy exist regarding the pathogenicity of *Y*. *enterocolitica* BT1A [57,72], in birds however no case-reports related to disease caused by BT1A were found. This could either be an indication that *Y*. *enterocolitica* BT1A does not tend to be pathogenic in birds, or that only limited research has been conducted on the pathogenicity of *Y*. *enterocolitica* BT1A in birds.

On the contrary, all recovered serotypes of *Y*. *pseudotuberculosis*, with serotype I being the most encountered serotype in Europe [24,73,74], have been implicated in yersiniosis cases and outbreaks in birds and mammals, including humans [37,73–78], but have also been isolated from apparently healthy birds and mammals [23,24,37,79,80]. The absence of the *virF* plasmid-borne virulence gene in serotype III and V is potentially an indication of a decrease in virulence [80,81], however, loss of the pYV virulence plasmid during the isolation/purification-procedure cannot be ruled out [19,74]. Since none of the positive birds in our study were recaptured, no inference can be made whether these house sparrows were temporary carriers with the potential of eliminating the pathogen, whether the passerines were in the incubation phase of the disease or actually presented a wildlife-reservoir of *Y*. *pseudotuberculosis*.

The dominant feeding strategy of the local bird assemblage affected the presence of *Y*. *pseudotuberculosis* and *Y*. *enterocolitica* in opposite ways. As for the pathogenic *Y*. *pseudotuberculosis*, higher prevalence of these bacteria was detected when the local bird populations were dominated by granivorous species, such as the highly susceptible Fringillidae [22,82], which, by using similar foraging strategies could enhance faeco-oral transmission [45–48]. On the other hand, *Y*. *enterocolitica* BT1A was negatively influenced by the degree of granivory of local bird communities, which could be an indication that, at least for this *Yersinia* species, granivourous birds are less suitable hosts/carriers than birds with other feeding patterns [83,84]. With respect to the other *Yersinia* species, no relation with granivory was demonstrated, suggesting that other, potentially more abiotic factors drive the distribution and prevalence of these *Yersinia* species [29]. However, since the group “*Yersinia* species” most likely comprises various species, the effect of granivores on the species-group could be neutralized due to counteracting effects on the separate *Yersinia* species. We should also keep in mind that for all analyses, the density of the different bird species was not taken into account, nor were other animals that could potentially act as a reservoir, which could likewise alter disease-ecology [12].

The prevalence of *Yersinia enterocolitica* and other *Yersinia* species was highly affected by the average daily temperature, being more prevalent when temperature was lower. As was previously observed when comparing *Yersinia*-survival in soil and water at different temperatures [27], the increased survival at colder temperatures potentially increases the bacteria-load in the environment and subsequently the prevalence in faeces. No such an effect was observed for *Y*. *pseudotuberculosis*, however the low prevalence likely decreased the power of the statistical analyses and potentially obscured potential relationships between temperature and prevalence.

The amount of built-up area had various effects on the presence of *Yersinia*. At the landscape scale, *Y*. *pseudotuberculosis* tended to be more prevalent in suburban hosts, and to a lesser extent in urban ones, compared to rural individuals. Although not investigated in our study, previous research has demonstrated higher densities of urban exploiters in suburban and urban regions [2,6], which could enhance the pathogen transmission in these habitats. On the contrary, *Y*. *enterocolitica* BT1A tends to be less prevalent in suburban house sparrows. The higher prevalence observed in the more urban areas could, similarly as for *Y*. *pseudotuberculosis*, be related to the higher density of other urban exploiters [6,85]. In rural areas on the other hand, other animals such as rodents, hares and livestock [52,86,87], possibly contribute to an increased occurrence of *Y*. *enterocolitica* BT1A in the examined house sparrows. Nevertheless, further investigations are warranted, including different taxa, and taking densities of all potential host species into account.

At the scale of individual home ranges, *Y*. *enterocolitica* and other *Yersinia* species were shown to be less prevalent in more urbanized habitats. This could be explained by the lower permeability of the surfaces in the more urbanized habitats, from which water excess is lost through run-off and as such dry-up relatively faster compared to actual soil substance [27,28]. Since *Yersinia* species are known to have a higher survival in wet to damp soil [27] the prevalence will likely be higher in less urbanized local habitats. The SMI did not have an influence on the presence of *Y*. *pseudotuberculosis*, *Y*. *enterocolitica* or other *Yersinia* species, neither did these *Yersinia* isolates affect the SMI of the house sparrows. With respect to *Y*. *enterocolitica* BT1A and the environmental *Yersinia* spp. it has been suggested that these *Yersinia* species are part of the normal avian microbiota [24,31], which could explain the lack of effect on house sparrows SMI. Nevertheless, only limited research has been performed on the pathogenicity of *Y*. *enterocolitica* BT1A and environmental *Yersinia* species in birds. *Yersinia pseudotuberculosis* on the other hand is known to be pathogenic for Passerines, and as such, a bi-directional effect of SMI and *Y*. *pseudotuberculosis* was expected. The lack of effect in either direction could be due to the low prevalence of *Y*. *pseudotuberculosis* in our house sparrow populations. However, as [24] previously suggested, wild birds potentially are able to sustain *Y*. *pseudotuberculosis* at low levels, without clinical signs, developing acute disease when exposed to stressful conditions.

The SMI was observed to increase from the morning to the afternoon, probably related to overnight fasting [26,88] although this observation is not always apparent [89]. Regarding the effect of urbanization on house sparrow body condition, most studies have compared strongly urbanized with rural habitats, disregarding the suburban areas (e.g.[9–11,89]). In this study, no significant differences were observed between populations from rural and strongly urbanized habitats, however, individuals from suburban populations had a higher SMI compared to urban populations (and to a lesser extent rural ones). Body condition has earlier been associated with stress response and overall health [9,30], though environmental factors such as habitat coverage [8], predictability of food supply and quality [10], presence of predators [8] have been hypothesized to influence the body condition of the birds. Suburban habitats in Flanders are typically characterized by strongly connected hedges and bushes, which are generally considered good habitat for house sparrows, allowing for a higher foraging efficiency compared to more fragmented highly urbanized or rural habitats. Indeed, [8] found that suitable foraging and shelter sites are highly scattered in urban areas. In rural areas, shelter sites are more connected than in highly urbanized areas, but the presence of intensive-agricultural fields forces sparrows to occupy larger home-ranges, increasing the energy expenditure when patrolling the entire home range, and thus potentially decreasing the body condition [8].

In conclusion, we here show that the urbanization gradient affects body condition and pathogen occurrence, two determinants of pathogen infection dynamics, suggesting a potential impact of urbanization on avian health. When assessing the impact of urbanization on animal health and pathogen dynamics, information regarding the presence/absence and preferably also the density of other suitable hosts, the two-way interaction between pathogen and host, and various levels of urbanization including the suburban habitat is required in order to have a better understanding of how urbanization can have an impact on urban wildlife health and diseases.

## Supporting information

S1 Table Sampled house sparrow populations with indication of the percentage of Build-Up-area in the local and landscape scale and provision of information regarding presence or absence of *Y*. *pseudotuberculosis* and *Y*. *enterocolitica*. Abbreviations similar as in Fig 1.
(DOCX)

S1 Protocol (g)lmer codes in R. (XLSX)

S1 Dataset Dataset comprising the variables included in the (g)lmer. (CSV)

S2 Dataset Dataset comprising the variables used to calculate the SMI of the house sparrows. (CSV)
