# Supplementary material for: Effects of urbanization on host-pathogen interactions, using Yersinia in house sparrows as a model
Source: PLoS One. 2017 Dec 27;12(12):e0189509. doi: 10.1371/journal.pone.0189509 (PMC5744950; doi:10.1371/journal.pone.0189509)
Supplement: S1 Table — Abbreviations similar as in Fig 1. (DOCX) [file pone.0189509.s001.docx]

| Plot | Landscape %BU | Home-range %BU | *Y. pseudotuberculosis* | *Y. enterocolitica* BT1A: #positive/total |
| --- | --- | --- | --- | --- |
| Ghent (Gh)1 | 18.27 | 23.58 | Serotype I | 0/9 |
| Gh2 | 38.98 | 41.39 | Serotype I | 7/14 |
| Beervelde (Be)1 | 7.30 | 15.80 | - | 1/5 |
| Be2 | 4.62 | 10.52 | - | 9/13 |
| Kalken (Ka)1 | 4.62 | 20.88 | - | 3/8 |
| Ka2 | 3.74 | 10.67 | - | 8/14 |
| Hillegem (Hi)1 | 4.62 | 22.17 | - | 1/6 |
| Hi2 | 4.65 | 5.97 | - | 7/12 |
| Melsen (Mel)1 | 3.81 | 13.97 | - | 0/8 |
| Mel2 | 2.13 | 7.20 | - | 0/6 |
| Oudenaarde (Ou)1 | 9.68 | 10.03 | - | 1/10 |
| Ou2 | 17.17 | 14.63 | Serotype I | 1/6 |
| Antwerp (An)1 | 7.51 | 23.51 | - | 1/7 |
| An2 | 24.52 | 55.03 | - | 1/11 |
| Pulderbos (Pu)1 | 2.56 | 5.11 | - | 4/10 |
| Pu2 | 3.68 | 7.04 | - | 11/16 |
| Herenthout (He)1 | 2.72 | 27.83 | Serotype I | 0/11 |
| He2 | 1.91 | 1.72 | - | 0/5 |
| Lint (Li)1 | 4.30 | 16.91 | - | 2/7 |
| Li2 | 11.79 | 31.61 | - | 3/7 |
| Mechelen (Me)1 | 22.14 | 27.37 | - | 4/11 |
| Me2 | 16.58 | 6.28 | - | 2/10 |
| Ruisbroek (Ru)1 | 3.09 | 5.95 | - | 0/9 |
| Ru2 | 6.09 | 19.97 | - | 2/4 |
| Leuven (Le)1 | 16.97 | 14.02 | - | 4/11 |
| Le2 | 27.90 | 33.75 | - | 6/9 |
| Wezemaal (We)1 | 4.69 | 3.28 | - | 0/6 |
| We2 | 5.31 | 30.83 | Serotype V | 1/9 |
| Houwaart (Ho)1 | 1.66 | 17.13 | Serotype III | 3/9 |
| Ho2 | 1.50 | 10.12 | - | 2/8 |
| Kerkom (Ke)1 | 1.99 | 14.32 | - | 2/11 |
| Ke2 | 1.82 | 7.16 | - | 6/10 |
| Tienen (Ti)1 | 10.86 | 7.73 | Serotype II | 6/12 |
| Ti2 | 17.85 | 27.20 | - | 2/15 |
| Overijse (Ov)1 | 8.99 | 3.71 | - | 2/5 |
| Ov2 | 8.23 | 39.41 | - | 1/5 |

**S1 Table. Sampled house sparrow populations, indicating the percentage of Build-Up-area in the local and landscape scale and providing information regarding presence or absence of *Y. pseudotuberculosis* and *Y. enterocolitica.***

Built-Up area = the percentage of Built-Up structures within a specified area:

>10% = urban (based on the landscape spatial scale)

5-10% = suburban (based on the landscape spatial scale)

<5% = rural (based on the landscape spatial scale)

Built-Up structure = a sustainable construction that encloses a space accessible for humans” (e.g. houses, garages, municipal buildings)
